# Artificial intelligence generated 3D body composition predicts dose modifications in patients undergoing neoadjuvant chemotherapy for rectal cancer

**DOI:** 10.1007/s00432-025-06219-5

**Published:** 2025-05-16

**Authors:** Alex Besson, Ke Cao, Ahmed Mardinli, Lara Wirth, Josephine Yeung, Rory Kokelaar, Peter Gibbs, Fiona Reid, Justin M. Yeung

**Affiliations:** 1 Department of Surgery - Western Precinct, The University of Melbourne, Melbourne, VIC Australia; 2 Melbourne Academic Centre for Health, North Melbourne, Melbourne, VIC Australia; 3 Western Health (Department of Colorectal Surgery), Footscray Hospital, Melbourne, VIC Australia; 4 Walter and Eliza Hall Institute, Parkville, Melbourne, VIC Australia; 5 Western Health (Department of Medical Oncology), Footscray Hospital, Melbourne, VIC Australia

**Keywords:** Rectal cancer, Chemotherapy, Dose modification, Body composition, Artificial intelligence

## Abstract

**Purpose:**

Chemotherapy administration is a balancing act between giving enough to achieve the desired tumour response while limiting adverse effects. Chemotherapy dosing is based on body surface area (BSA). Emerging evidence suggests body composition plays a crucial role in the pharmacokinetic and pharmacodynamic profile of cytotoxic agents and could inform optimal dosing. This study aims to assess how lumbosacral body composition influences adverse events in patients receiving neoadjuvant chemotherapy for rectal cancer.

**Methods:**

A retrospective study (February 2013 to March 2023) examined the impact of body composition on neoadjuvant treatment outcomes for rectal cancer patients. Staging CT scans were analysed using a validated AI model to measure lumbosacral skeletal muscle (SM), intramuscular adipose tissue (IMAT), visceral adipose tissue (VAT), and subcutaneous adipose tissue volume and density. Multivariate analyses explored the relationship between body composition and chemotherapy outcomes.

**Results:**

242 patients were included (164 males, 78 Females), median age 63.4 years. Chemotherapy dose reductions occurred more frequently in females (26.9% vs. 15.9%, *p* = 0.042) and in females with greater VAT density (-82.7 vs. -89.1, *p* = 0.007) and SM: IMAT + VAT volume ratio (1.99 vs. 1.36, *p* = 0.042). BSA was a poor predictor of dose reduction (AUC 0.397, sensitivity 38%, specificity 60%) for female patients, whereas the SM: IMAT + VAT volume ratio (AUC 0.651, sensitivity 76%, specificity 61%) and VAT density (AUC 0.699, sensitivity 57%, specificity 74%) showed greater predictive ability. Body composition didn’t influence dose adjustment of male patients.

**Conclusion:**

Lumbosacral body composition outperformed BSA in predicting adverse events in female patients with rectal cancer undergoing neoadjuvant chemotherapy.

**Supplementary Information:**

The online version contains supplementary material available at 10.1007/s00432-025-06219-5.

## Introduction

Neoadjuvant chemoradiotherapy (CRT) is the current standard of care for locally advanced rectal cancer (LARC) treated with curative intent. With the increasing adoption of total neoadjuvant therapy (TNT), additional pre-operative chemotherapy is administered in addition to traditional long-course CRT regimens (Bedrikovetski et al. 2024). Dosing for most chemotherapy drugs is based on body surface area (BSA), a poor indicator of drug metabolism and clearance. BSA also doesn’t account for the substantial variations in body composition and relative volumes of lean body mass (LBM) or adipose tissue that can occur in people of the same weight. There is increasing evidence that body composition assessment could be used to refine chemotherapy dosing, reducing toxicity without compromising efficacy (da Silva Dias et al. 2022).

Skeletal muscle (SM) volume has been shown to correlate with chemotherapy adverse events, (Ali et al. 2016) however the published literature is inconsistent, in part due to varied methodology in assessing body composition. (Drami et al. 2021) Commonly used chemotherapy agents in rectal cancer treatment include Oxaliplatin, a fluoropyrimidine (either 5-fluorouracil [5-FU] or capecitabine), with irinotecan now included in some TNT regimens. Oxaliplatin is a complex platinum based molecule with both lipophilic and hydrophilic properties and distribution in adipose tissue predominates whilst fluoropyrimidines are hydrophilic with greater uptake in SM (Aslani et al. 2000; Lévi et al. 2000). These differing qualities suggest that the variability in body composition, assessing both muscle mass and adipose tissue, which will vary for a given BSA could usefully inform the optimal dosing of both agents.

Computed tomography (CT) derived body composition analysis has been widely used over the last decade, progressing from manual and time consuming 2D based assessment to automated artificial intelligence (AI) derived 3D volumetric analysis (Cao et al. 2023, 2025). With machine learning software, body composition can now be rapidly acquired from routine staging CT scans, making this information available to clinicians for each patient.

The aim of this study was to utilise AI mediated 3D body composition to determine the impact of SM and of adipose tissue on chemotherapy adverse effects with a goal of informing body composition-based dosing guidelines for chemotherapy agents.

## Methods

A retrospective study of patients with LARC treated between February 2013 to March 2023 was conducted examining chemotherapy adverse events associated with neoadjuvant treatment, focusing on the need for dose reductions and treatment cessation due to toxicity. Ethics approval was granted by Western Health (WH) Ethics Committee (Project number QA.2018.74).

### Patient selection

Rectal cancer patients treated at WH were identified from the Australian Comprehensive Cancer Outcomes and Research Database (ACCORD), a prospective clinical registry for colorectal cancer patients within Victoria, Australia. Patients were included in the study if they had a histological diagnosis of rectal adenocarcinoma and were treated with curative intent, which included neoadjuvant chemotherapy.

### Data collection

Patient demographics, tumour characteristics, treatment administered, and oncological and surgical outcome data was collected from the ACCORD database and cross referenced with WH electronic medical records (EMR). Neoadjuvant adverse event data were obtained from the WH EMR, including any adverse event from chemotherapy treatment that resulted in a dose reduction or early cessation. Chemotherapy toxicity was considered as an any grade of toxicity, as defined by the Common Terminology Criteria for Adverse Events (CTCAE, version 5), associated with cytotoxic medications. Staging CT scans were retrieved for all patients and Digital Imaging and Communications in Medicine files were downloaded and used for body composition analysis with an in-house validated AI software(Cao et al. 2024).

## Neoadjuvant oncological treatment

Neoadjuvant treatment for rectal cancer at WH involves CRT, in which infusional 5-FU or capecitabine is given alongside long course radiotherapy (50 Gy in 25 fractions). Treatment dosing is based on BSA. Since 2020, selected patients with LARC have been considered for TNT, with the addition of neoadjuvant chemotherapy, either four cycles of CAPOX (Capecitabine & Oxaliplatin) or six cycles of FOLFOX (5-FU, Leucovorin & Oxaliplatin). The use of TNT and which oncological agent/regimen was decided by a multidisciplinary team meeting and the treating medical oncologist.

### Body composition measurement

Body composition analysis from each patient’s lumbosacral region was derived from an AI generated analysis of staging CT scans prior to neoadjuvant treatment. Data on tissue volume (cm^3^) and average radiodensity (Hounsfield units, HU), for body composition including SM, intramuscular adipose tissue (IMAT), visceral adipose tissue (VAT) and subcutaneous adipose tissue (SAT) were collected with a pre-trained and validated in-house AI segmentation model (Cao et al. 2024). Three-dimensional body composition for each tissue type was determined by assessing axial CT scan slices within the lumbosacral region as determined by a trained investigator (AB & JY). Tissue volume was determined by multiplying the area of tissue present in each axial slice by the CT slice thickness. Average tissue radiodensity (HU) was determined by the sum of the mean radiodensity in each axial slice, for each tissue compartment, divided by the number of slices in the lumbosacral region. We explored the relationship of SM to adipose tissue to develop a novel scoring index based on volume ratios (SM: VAT + IMAT). Patients were excluded from the study if their CT scan could not be accurately analysed, this included poor image quality (e.g. artifact or interference) or if there was extension of soft tissue outside the captured CT image.

### Statistical analysis

Descriptive statistics were used for baseline patient demographics to compare between groups. For continuous variables, median values and interquartile range (IQR) were compared using the Mann Whitney U Test, whilst categorical values were assessed using Fisher’s exact test. A multivariate analysis was performed to identify the relationship between body composition and chemotherapy dose modification. Receiver operating characteristics (ROC) analyses were performed for each body composition metric as well as age, BSA and BMI to calculate the area under the curve (AUC). The AUC value was used to demonstrate each parameters association with chemotherapy dose modification and optimal cutpoint values from the ROC curve were identified using the Liu method. All statistical analysis was performed using STATA (version BE 18.0).

## Results

A total of 242 patients (Male 164, 67.8%) received curative treatment for rectal cancer at WH during the study period. Key patient demographics and oncological treatment are presented in Table 1. 33 patients received TNT, whilst the remaining 209 patients were treated with long course chemoradiotherapy.

**Table 1:** Key patient demographics and neoadjuvant treatment

Parameter	Male (*n* = 164)	Female (*n* = 78)	*p*-value
Age at diagnosis	63.6 (54.4–72.2)	63.0 (55.3–74.5)	0.192
Smoking status - Non-smoker - Current smoker - Ex-smoker	58 (35.4%) 45 (27.4%) 61 (37.2%)	48 (62.3%) 10 (13.0%) 19 (24.7%)	< 0.001^*^
Diabetic status - Non-diabetic - T1DM - T2DM	130 (79.3%) 0 (0%) 34 (20.7%)	60 (79.9%) 1 (1.3%) 17 (21.8%)	0.399
ASA^a^ score − 1 − 2 − 3 − 4 - Unknown	8 (4.9%) 79 (48.2%) 70 (42.7%) 2 (1.2%) 5 (3.1%)	3 (3.9%) 39 (50%) 35 (44.9%) 1 (1.3%) 0 (0%)	0.683
ECOG^b^ score − 0 − 1 − 2 − 3 − 4	130 (79.3%) 25 (15.2%) 8 (4.9%) 1 (0.6%) 0 (0%)	59 (75.6%) 15 (19.2%) 4 (5.1%) 0 (0%) 0 (0%)	0.810
Neoadjuvant chemotherapy - FOLFOX - CAPOX	*n* = 21 (12.8%) 7 (33.3%) 14 (66.7%)	*n* = 12 (15.4%) 4 (33.3%) 8 (66.7%)	1.000
Neoadjuvant CRT^c^ − 5-Fluorouracil - Capecitabine	*n* = 161 (98.2%) 69 (42.9%) 92 (57.1%)	*n* = 76 (97.4%) 38 (50%) 38 (50%)	0.329
Chemotherapy toxicity	39.6%	59.0%	0.006^*^
Chemotherapy dose adjustment	15.9%	26.9%	0.042^*^

a: American Society of Anaesthesiologists, b: Eastern Cooperative Oncology Group, c: Chemoradiotherapy

Male patients were more likely to smoke tobacco (Male 27.4% vs. Female 13.0%, *p* < 0.001) whilst remaining patient demographics showed no statistical difference between genders. Body composition varied greatly between male and female patients (Supplementary Table 1) with significant differences for all volumetric body composition measurements between genders, except for IMAT. Male patients had greater SM (6823 vs. 4807cm^3^, *p* < 0.001) and VAT (3901 vs. 2524cm^3^, *p* < 0.001) volumes with greater SM (40.5 vs. 38.9 HU, *p* = 0.042) and SAT (-97.2 vs. -101.9 HU, *p* < 0.001) density; conversely female patients had greater SAT volume (7827 vs. 5604cm^3^, *p* < 0.001). No difference in IMAT and VAT density was identified between genders.

As shown in Table 1, female patients were more likely to require chemotherapy dose modification during treatment (Male 15.9% vs. Female 26.9%, *p* = 0.042) with greater rates of any grade chemotherapy toxicity (Male 39.6% vs. Female 59.0%, *p* = 0.006).

Gender specific body composition comparison for patients with and without dose adjustments are detailed in Table 2. SM: IMAT + VAT volume ratio was higher in female patients requiring dose adjustment (1.99 vs. 1.36, *p* = 0.042) whilst a greater VAT volume (3080 vs. 1983cm^3^, *p* = 0.033) was associated with fewer dose adjustments for females in our cohort. Increased IMAT (-55.2 vs. -57.3 HU, *p* = 0.047) and VAT (-82.7 vs. -89.1 HU, *p* = 0.007) density was observed in female patients with dose adjustments. There was no body composition difference in male patients with dose adjustments compared to those without dose adjustments.

**Table 2:** Body composition dose modification vs. no- dose modification

Parameters	Male (*n* = 164)			Female (*n* = 78)		
	Dose modification (*n* = 26)	No dose modification (*n* = 138)	*p*-value	Dose modification (*n* = 21)	No dose modification (*n* = 57)	*p*-value
BSA^a^ (m^2^)	1.93 (1.86–2.11)	1.92 (1.79–2.08)	0.65	1.69 (1.59–1.83)	1.75 (1.65–1.90)	0.16
BMI^b^ (kg/m^2^)	26.2 (24.7–29.8)	27.2 (23.6–30.7)	0.93	25.6 (21.9–30.9)	29.1 (25.9–31.6)	0.08
SM^c^ volume(cm^3^)	6762 (6153–8031)	6835 (5884–7519)	0.46	4607 (4164–4947)	4947 (4263–5609)	0.09
SM density (HU)	43.0 (36.2–46.4)	40.5 (34.3–45.5)	0.49	36.5 (31.7–46.3)	39.0 (32.4–42.4)	0.7
IMAT^d^ volume (cm^3^)	471 (355–612)	447 (317–637)	0.76	354 (312–488)	438 (343–608)	0.23
IMAT density (HU)	-57.8 (-60.2–55.3)	-56.3 (-58.6–54.7)	0.25	-55.2 (-56.8–54.8)	-57.3 (-60.3–54.7)	0.047^*^
VAT^e^ volume (cm^3^)	3740 (2734–6345)	3950 (2496–5740)	0.74	1983 (1572–2657)	3080 (1834–4443)	0.033^*^
VAT density (HU)	-88.3 (-93.8–84.8)	-88.7 (-92.8–83.6)	0.89	-82.7 (-88.3–79.8)	-89.1 (-94.0–83.4)	0.007^*^
SAT^f^ volume (cm^3^)	5604 (3933–7696)	5015 (3530–7204)	0.57	7743 (5156 − 1033)	8176 (6523–11274)	0.12
SAT density (HU)	-96.6 (-98.9–90.1)	-97.2 (-101.1–92.2)	0.29	-99.3 (-106.2–93.9)	-102.0 (-106.1–98.3)	0.35
Muscle: Fat^g^ (cm^3^)	0.65 (0.55–0.85)	0.70 (0.50-1)	0.69	0.48 (0.40–0.69)	0.40 (0.33–0.50)	0.062
Muscle: IMAT + VAT^h^ (cm^3^)	1.56 (1.16–1.91)	1.50 (1.07–2.42)	0.77	1.99 (1.62–2.56)	1.36 (0.99–2.16)	0.042^*^
Muscle: IMAT^i^ (cm^3^)	15.5 (11.6–20.0)	15.2 (10.6–20.3)	0.95	12.1 (9.27–14.3)	11.3 (7.86–14.8)	0.51

a: Body Surface Area, b: Body Mass Index, c: Skeletal Muscle, d: Intramuscular Adipose Tissue, e: Visceral Adipose Tissue, f: Subcutaneous Adipose Tissue, g: Skeletal muscle to total fat volume ratio, h: Skeletal muscle to intramuscular adipose tissue and visceral adipose tissue volume ratio, i: Skeletal muscle to intramuscular adipose tissue volume ratio

**Fig. 1:**
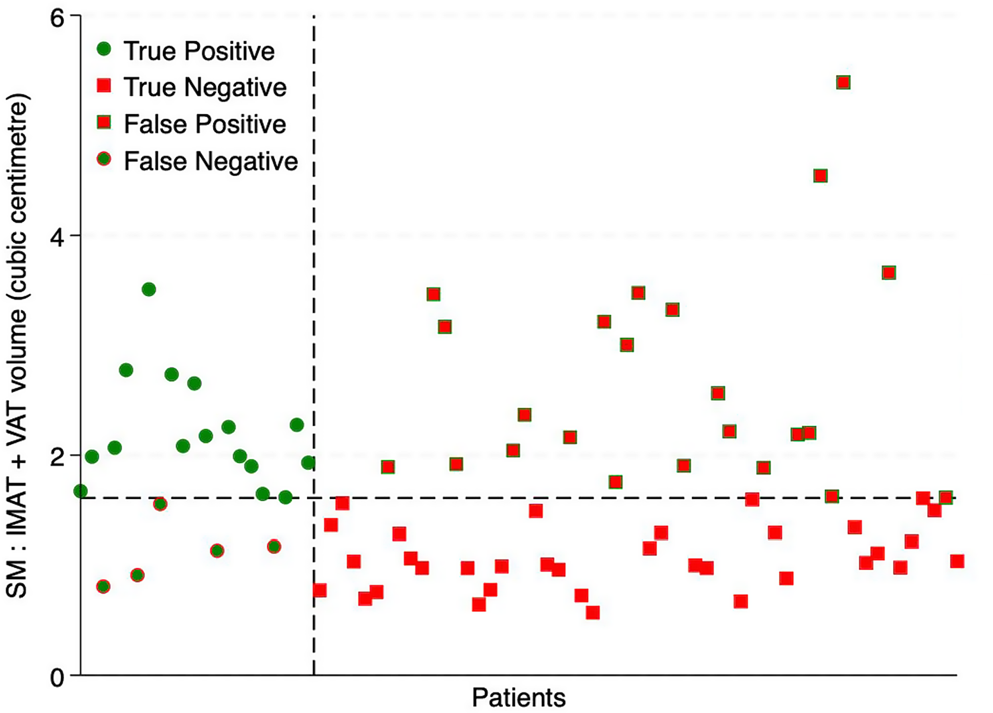
SM: IMAT + VAT volume prediction of dose modification for female patients

**Fig. 2:**
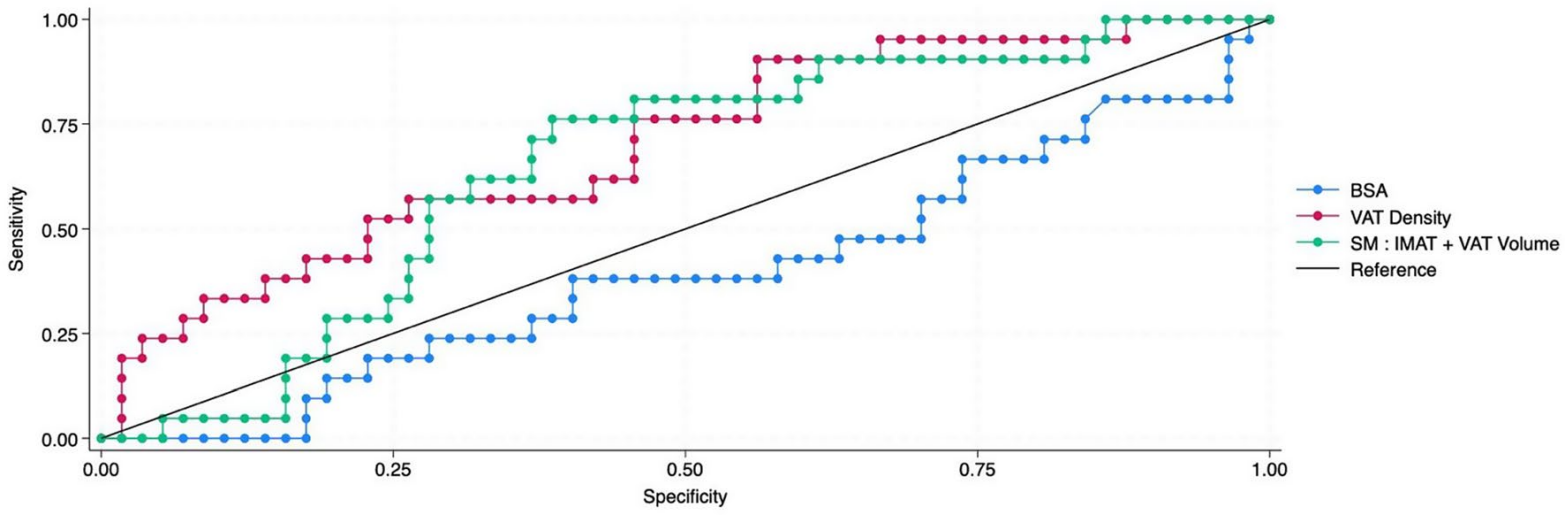
Female chemotherapy dose adjustment ROC Curve: BSA compared to VAT density & SM: IMAT + VAT volume

Each body composition parameter underwent ROC analysis to determine the AUC value to evaluate its ability to discriminate between the likelihood of dose adjustments (Table 3). For female patients, BSA provided little predictive power (AUC 0.397, sensitivity 38% and specificity 60%) whilst VAT density (AUC 0.699, sensitivity 57% and specificity 74%) and SM: IMAT + VAT volume ratio (Fig. 1) (AUC 0.651, sensitivity 76% and specificity 61%) showed the greatest association with dose adjustments (Fig. 2).

**Table 3:** ROC and AUC with optimal cutpoints, sensitivity and specificity

Parameter	Male				Female			
	AUC	Optimal cutpoint	Sensitivity (%)	Specificity (%)	AUC	Optimal cutpoint	Sensitivity (%)	Specificity (%)
BSA^a^ (m^2^)	0.523	1.88	69	42	0.397	1.82	38	60
BMI^b^ (kg/m^2^)	0.492	25.1	69	39	0.369	29.4	38	56
SM^c^ volume (cm^3^)	**0.549**	7314	43	70	0.376	4565	57	39
SM density (HU)	**0.549**	44.8	48	72	**0.528**	43.9	0.38	0.77
IMAT^d^ volume (cm^3^)	0.517	541	43	66	**0.41**	443	38	51
IMAT density (HU)	0.428	-56.1	43	54	**0.648**	-56	0.81	0.59
VAT^e^ volume (cm^3^)	0.52	2972	70	37	0.342	1547	81	21
VAT density (HU)	0.491	-99.3	52	53	**0.699**	-83.7	57	74
SAT^f^ volume (cm^3^)	**0.54**	5214	65	52	**0.386**	7734	52	46
SAT density (HU)	**0.567**	-96.8	57	55	**0.57**	-97.5	43	81
SM: Adipose^g^ (cm^3^)	0.473	0.6	70	39	**0.638**	0.44	67	67
SM: IMAT + VAT^h^ (cm^3^)	0.483	1.39	0.65	0.46	**0.651**	1.61	76	61
SM: IMAT^i^ (cm^3^)	0.508	13.8	65	45	**0.549**	11.9	57	61

Body composition measurements with an AUC greater than BSA are highlighted in bold

a: Body Surface Area, b: Body Mass Index, c: Skeletal Muscle, d: Intramuscular Adipose Tissue, e: Visceral Adipose Tissue, f: Subcutaneous Adipose Tissue, g: Skeletal muscle to total fat volume ratio, h: Skeletal muscle to intramuscular adipose tissue and visceral adipose tissue volume ratio, i: Skeletal muscle to intramuscular adipose tissue volume ratio

## Discussion

Chemotherapy toxicity causes a significant morbidity and burden for patients with colorectal cancer. Moderate to severe toxicity was reported to occur in 45.7% of patients and often results in delayed treatment or early cessation of cytotoxic therapy (Han et al. 2024). Numerous risk factors associated with chemotherapy toxicity have been described but there is growing evidence that body composition plays a significant role in the determination of toxicity (Cao et al. 2025).

In patients undergoing standard neoadjuvant therapy for rectal cancer, we found evidence chemotherapy toxicity was more frequent in female patients. Dose reduction or early cessation was required for 26.9% of female patients and 15.9% of male patients (*p* = 0.042). This gender based difference is consistent with the findings of a recent meta-analysis by Han et al (Han et al. 2024). Body composition varied between genders as previously reported by others(Bredella 2017) with greater SM (6823 vs. 4807cm^3^, *p* < 0.001) and VAT volume (3901 vs. 2523cm^3^, *p* < 0.001) seen in male patients, whilst greater SAT volume (7827 vs. 5280cm^3^, *p* < 0.001) was observed in female patients. Body composition variation between genders have been proposed to contribute to the well-established gender-based differences in chemotherapy toxicity profiles (Baraibar et al. 2023).

In our female cohort, increased VAT density (-82.7 vs. -89.1 HU, *p* = 0.007) and reduced VAT volume (1983 vs. 3080cm^3^, *p* = 0.033) were both associated with an increased incidence of dose modification. Increased adipose density can reflect inflammatory or metabolic changes that can occur because of the proinflammatory cytokine response from the tumour itself or because of fibrosis following periods of weight loss (Pellegrini et al. 2023; Cheng et al. 2022; Tuomisto, Mäkinen, and Väyrynen 2019). A significant decrease in weight prior to diagnosis of rectal cancer and increased radiodensity of adipose tissue are both associated with poorer overall survival, however, the relationship with chemotherapy toxicity is poorly established (Pellegrini et al. 2022; Charette et al. 2019; Walter et al. 2016).

Low VAT volume for female patients was associated with increased dose modifications. We believe VAT volume in this context could be used as a marker of systemic malnutrition with low volume indicating body composition changes due to cancer cachexia. We found that female patients with low VAT volumes were more likely to have concurrent sarcopenia (42% vs. 19%, *p* = 0.038) than females with greater VAT volume. Sarcopenia has been defined as the lowest quartile of SM volume; we have used this definition as there is no current internationally validated threshold to define sarcopenia from 3D volumetric body composition analysis. Previous 2D body composition assessment of our institutional cohort identified 62.5% of patients as being sarcopenic, which mirrors our current incidence of sarcopenia within this study (Besson et al. 2021).

Gold standard dosing of chemotherapy agents is calculated based on BSA, however, this commonly leads to suboptimal therapeutic dosing. Li et al. showed, in a prospective multicentre observational trial, that plasma levels of 5-FU were subtherapeutic in 60.6% of patients, supratherapeutic in 19.1%, with only the remaining 20.3% of patients being within the desired therapeutic range (Li et al. 2023). There are multiple explanations for these variations including the gender based metabolic function differences, in particular plasma clearance of 5-FU which is 26% slower in female patients (Port et al. 1991). Body composition differences between genders could also explain the variable drug levels experienced by patients as shown by the increased rates of toxicity experienced by our female patients, (Male 39.6% vs. Female 59.0%, *p* = 0.006), similar to previously reported studies in the literature (Cao et al. 2023; Han et al. 2024).

Two-dimensional CT body composition analysis of an axial CT image at the mid-L3 vertebra had been considered the gold standard of body composition analysis (Prado et al. 2008). However, recent development and validation of AI algorithms capable of auto-segmentation and volumetric body composition analysis can provide reproducible and accurate 3D body composition reports which outperform 2D segmentation. There is evidence to support the use of LBM over BSA for dosing many chemotherapy agents (Ali et al. 2016; Cao et al. 2023) with increased SM volume shown to improve rates of chemotherapy completion (Cao et al. 2023). The time-consuming nature of 2D manual segmentation make body composition analysis impractical for clinical application, however, with the advent of AI automated segmentation this barrier has been substantially reduced.

Body composition derived dosing regimens have been increasingly reported in the literature; however, this is yet to impact routine clinical practice. Prado et al. have shown that a 5-FU dose > 20 mg/Kg of LBM is associated with an increased risk of developing toxicity (OR 16.75, *p* = 0.013) (Prado et al. 2007), whilst Ali et al. identified an Oxaliplatin dose of 3.09 mg/Kg of LBM where patients receiving a dose greater than this had a 44% risk of treatment modification (Ali et al. 2016).

We found that BSA for female patients was very poorly correlated with treatment modification (AUC value of 0.397) as compared with other body composition metrics (Table 3). The two greatest predictors were VAT density and SM: VAT + IMAT volume ratio with an AUC value of 0.699 and 0.651 respectively. A cutpoint value of 1.61 for SM: VAT + IMAT volume ratio resulted in a sensitivity and specificity of 76% and 61% (Fig. 1). Values greater than this cutpoint had a relative risk of 3.2 (95% CI 1.3–7.9, *p* = 0.01) for requiring dose modification. Body composition volumetric ratios were explored as a novel approach to 3D body composition analysis; to our knowledge this has not previously been reported in the literature and provides promising initial results which require further investigation to assess its role as a means of dosing chemotherapy.

Fluoropyrimidine-based chemotherapy sides effects are well documented and include, haematological and gastrointestinal complications (Knikman et al. 2021). Dependent on severity, these side effects can cause significant reduction in quality of life and result in treatment interruptions or early cessation (Knikman et al. 2021). Lișcu et al. found that treatment delays of > 3 days were associated with decreased overall survival (hazard ratio 5.89, *p* < 0.001) whilst delays of > 2 days were associated with decreased rates of disease free survival (82.2 vs. 50.5%, *p* < 0.001) (Lișcu et al. 2024). The use of body composition dosing guidelines offers a potential way of personalising chemotherapy dosing with the aim of reducing chemotherapy toxicity and improving patient outcomes.

Despite these findings, there were some limitations within our study. Some clinical data was collected retrospectively from the hospital EMR; in particular details of toxicities due to chemotherapy. However, the ACCORD database is prospectively maintained where extensive patient data and complications were collected. Chemotherapy toxicity grade was not reported as sufficient documentation was not available in all cases to make an accurate CTCAE grade classification, for this reason we have grouped all toxicity grades together. The impact of radiotherapy on haematological toxicity was unable to be accounted for and may contribute to the small number of haematological toxicity events recorded during chemoradiotherapy.

## Conclusion

Differences in gender specific treatment modification is multifactorial; there is increasing evidence to show that body composition differences play a crucial role. Given the gender disparities in chemotherapy toxicity outcomes, a new approach to chemotherapy dosing needs to be considered. Body composition could offer an individualised and nuanced approach to dosing chemotherapy agents thereby reducing treatment modification and improving chemotherapy completion rates.

## Electronic supplementary material

Below is the link to the electronic supplementary material.

Supplementary Material 1

## Data Availability

Data is supplied within the manuscript and supplementary information. If any further access to raw data is required please contact the corresponding author.
