# Supplementary material for: Artificial intelligence generated 3D body composition predicts dose modifications in patients undergoing neoadjuvant chemotherapy for rectal cancer
Source: J Cancer Res Clin Oncol. 2025 May 16;151(5):168. doi: 10.1007/s00432-025-06219-5 (PMC12084224; doi:10.1007/s00432-025-06219-5)
Supplement: Supplementary file 1 — Supplementary Material 1 [file 432_2025_6219_MOESM1_ESM.docx]

Artificial intelligence generated 3D body composition predicts dose modifications in patients undergoing neoadjuvant chemotherapy for rectal cancer.

Journal of cancer research and surgical oncology

Authorship:

Alex Besson^1,2^ BBiomedSc, MD

Ke Cao^1^ MSc, PhD

Ahmed Mardinli^1^ BBiomed, MD

Lara Wirth^1^ MBiomedSc

Josephine Yeung^1^ BPharm, MRPharmS

Rory Kokelaar^1,3^ MBBS MA MEd PhD FRCS

Peter Gibbs^4,5^ MBBS FRACP MD

Fiona Reid^1,3^ BmedSci BMBS MSurgEd FRACS

Justin M Yeung^1,3^ DM, FRCSEd (Gen Surg), FRACS

1. The University of Melbourne (Department of Surgery - Western Precinct), Melbourne, Victoria, Australia
2. Melbourne Academic Centre for Health, North Melbourne, Victoria, Australia
3. Western Health (Department of Colorectal Surgery), Footscray Hospital, Melbourne, Victoria, Australia
4. Walter and Eliza Hall Institute, Parkville, Melbourne, Victoria, Australia
5. Western Health (Department of Medical Oncology), Footscray Hospital, Melbourne, Victoria, Australia

Correspondence

Email: justin.yeung@unimelb.edu.au

Supplementary Table 1: Gender specific body composition summary

| Parameter | Male | Female | P-value |
| --- | --- | --- | --- |
| BSA (m^2^) | 1.92 (1.80-2.08) | 1.75 (1.62-1.89) | <0.001 |
| BMI (kg/m^2^) | 26.9 (23.6-30.4) | 28.7 (25.0-31.2) | 0.098 |
| SM volume (cm^3^) | 6823 (5886-7606) | 4807 (4215-5515) | <0.001 |
| SM Density (HU) | 40.5 (34.5-46.3) | 38.9 (32.2-44.0) | 0.042 |
| IMAT volume (cm^3^) | 452 (319-637) | 422 (319-598) | 0.649 |
| IMAT Density (HU) | -56.6 (-58.8--54.9) | -56.4 (-60.1--54.7) | 0.993 |
| VAT volume (cm^3^) | 3901 (2565-5782) | 2524 (1665-3981) | <0.001 |
| VAT Density (HU) | -88.7 (-92.8--83.7) | -87.9 (-92.9--82.3) | 0.577 |
| SAT volume (cm^3^) | 5604 (3933-7696) | 7827 (6317-10473) | <0.001 |
| SAT Density (HU) | -97.2 (-100.7--91.0) | -101.9 (-106.2-97.4) | <0.001 |
| Muscle:Adipose (cm^3^) | 0.69 (0.51-0.97) | 0.42 (0.33-0.55) | <0.001 |
| Muscle:IMAT+VAT (cm^3^) | 1.52 (1.08-2.29) | 1.61 (1.02-2.19) | 0.804 |
| Muscle:IMAT (cm^3^) | 15.2 (10.8-20.2) | 11.3 (7.86-14.8) | <0.001 |
